# Feeding on *Beauveria bassiana*-treated *Frankliniella occidentalis* causes negative effects on the predatory mite *Neoseiulus barkeri*

**DOI:** 10.1038/srep12033

**Published:** 2015-07-08

**Authors:** Shengyong Wu, Yulin Gao, Xuenong Xu, Dengjie Wang, Juan Li, Haihong Wang, Endong Wang, Zhongren Lei

**Affiliations:** 1 State Key Laboratory for Biology of Plant Diseases and Insect Pests, Institute of Plant Protection, Chinese Academy of Agricultural Sciences, Beijing 100193, P.R. China

## Abstract

The entomopathogenic fungus *Beauveria bassiana* and the predatory mite *Neoseiulus barkeri* are both potential biocontrol agents for their shared host/prey *Frankliniella occidentalis*. The combination of the two agents may enhance biological control of *F. occidentalis* if the fungus does not negatively affect *N. barkeri*. This study evaluated the indirect effects of *B. bassiana* strain SZ-26 on *N. barkeri* mediated by *F. occidentalis* using the age-stage, two-sex life table. When fed on the first instar larvae of *F. occidentalis* that had been exposed for 12 h to the SZ-26 suspension, the developmental time of preadult *N. barkeri* was significantly longer, and the longevity and fecundity were significantly lower than that of *N. barkeri* fed on untreated *F. occidentalis*. The mean generation time (*T*), net reproductive rate (*R*_*0*_), finite rate of increase (λ), intrinsic rate of natural increase (*r*_*m*_) and predation rates were correspondingly affected. The data showed that *B. bassiana* has indirect negative effects on *N. barkeri* population dynamics via influencing their prey *F. occidentalis* larvae, which indicates that there is a risk in combining *B. bassiana* with *N. barkeri* simultaneously for the biocontrol of *F. occidentalis*. The probable mechanism for the negative effects is discussed.

The western flower thrips, *Frankliniella occidentalis* (Pergande) (Thysanoptera: Thripidae), is a major insect pest of ornamental plants and vegetables that causes extensive economic losses in many crops worldwide^1^,^2^,^3^,^4^,^5^,^6^. The intensive use of conventional synthetic insecticides has led to widespread resistance^7^. Therefore, there has been an increasing interest in managing *F. occidentalis* populations using biological control agents. The predatory mite *Neoseiulus barkeri* (*Amblyseius barkeri*) Hughes (Acarina: Phytoseiidae) is a specialist predator of *F. occidentalis* that has been used as an alternative for *F. occidentalis* management on a variety of plants^8^,^9^,^10^,^11^. However, control efficiency is limited because the predatory mites prey primarily on only two larval stages of thrips, and because success in capturing prey depends largely on the size and feeding status of the predator^12^. Control with predatory mites alone is not generally effective when there is high density of *F. occidentalis* in a crop.

Entomopathogenic fungi have been successfully developed worldwide as biological control agents for many arthropod pests, including several species of thrips^13^,^14^,^15^. The entomopathogenic fungus *Beauveria bassiana* (Balsamo) Vuillemin (Hypocreales: Cordycipitaceae) has potential for managing *F*. *occidentalis*. While commercial development of thrips-active isolates of these fungi has been limited, recent studies in China screened several isolates of *B. bassiana* that are highly virulent against *F. occidentalis* and showed their potential as biological control agents^16^,^17^,^18^,^19^. Recently, in our bioassay experiments, strain SZ-26 was screened from 30 isolates of *B. bassiana* and identified as one of the most virulent strains, causing 97% mortality to *F. occidentalis* adults at 85 viable conidia per mm^2^, while it showed no virulence to *N. barkeri* when sprayed directly on the mites^20^. Although the fungus works rapidly and kills large numbers of adult *F. occidentalis* via infection in the short term, pathogenicity to immature stages of *F. occidentalis* is reduced because fungal conidia are shed with the exuvium following ecdysis^14^,^21^. Uninfected immature *F. occidentalis* continue to propagate and damage crops.

Recently, an integrated control approach, including the use of multiple biocontrol agents for pest management has been of growing interest to biological control practitioners^22^,^23^,^24^,^25^,^26^. The combination of *N. barkeri* with *B. bassiana* is a promising control measure for maintaining *F*. *occidentalis* populations below economically damaging levels, and solving problems of chemical resistance. However, the potential benefits and hazards of using fungus and predators simultaneously have not yet been widely evaluated. Multiple biological control species may act synergistically, additively or antagonistically^27^, and the potential for integrated pest management (IPM) may be compromised if the predators are susceptible to *B. bassiana* when the conidia are directly applied, or if the predators are indirectly affected via feeding on contaminated surfaces or infected prey.

The majority of previous studies evaluated the effects of pathogens on predators by exposing them directly to residues, or by short term topical application and then studying predator behavior, mortality and population abundance^16^,^26^,^28^,^29^,^30^,^31^. However, only a few studies were conducted to understand the effect of fungus infection on the physiology of the host and its indirect effect on predators by feeding behavior^32^. The quality and nutritional value of the prey may cause sub-lethal effects on predators^33^, such as changes in development time, consumption rate and life table parameters, which play an important role in determining the population growth rate. The objective of this study was to evaluate the effects of the *B. bassiana*-treated *F*. *occidentalis* on *N. barkeri* by studying the predator’s biological parameters.

## Results

### Life table

The development periods for each life stage, adult longevity, preoviposition period, and female fecundity of *N. barkeri* fed on *B. bassiana*-treated (treatment) and untreated (control) *F. occidentalis* larvae are shown in Table 1. Because the hatching of eggs and development of larvae are not affected by prey, the duration of the egg and larval stages of *N. barkeri* were not significantly different between the control and treatment. However, there was a significantly longer protonymph duration (*P* < 0.001), deutonymph duration (*P* < 0.001) and preadult period (*P* < 0.001) in the treatment than in the control. There were no differences between treatment and control groups in terms of adult pre-oviposition period (APOP). In contrast to the APOP, the total pre-oviposition period (TPOP) (from the beginning of the life table study to first egg production) was longer for the treatment than the control group (*P* < 0.001). Adult longevity (both female and male) was shorter (*P* < 0.001), and fewer eggs were produced (*P* < 0.001) in the treatment than the control group. The female adult sex ratio decreased and was less than 50% for the treatment.

**Figure 1:**
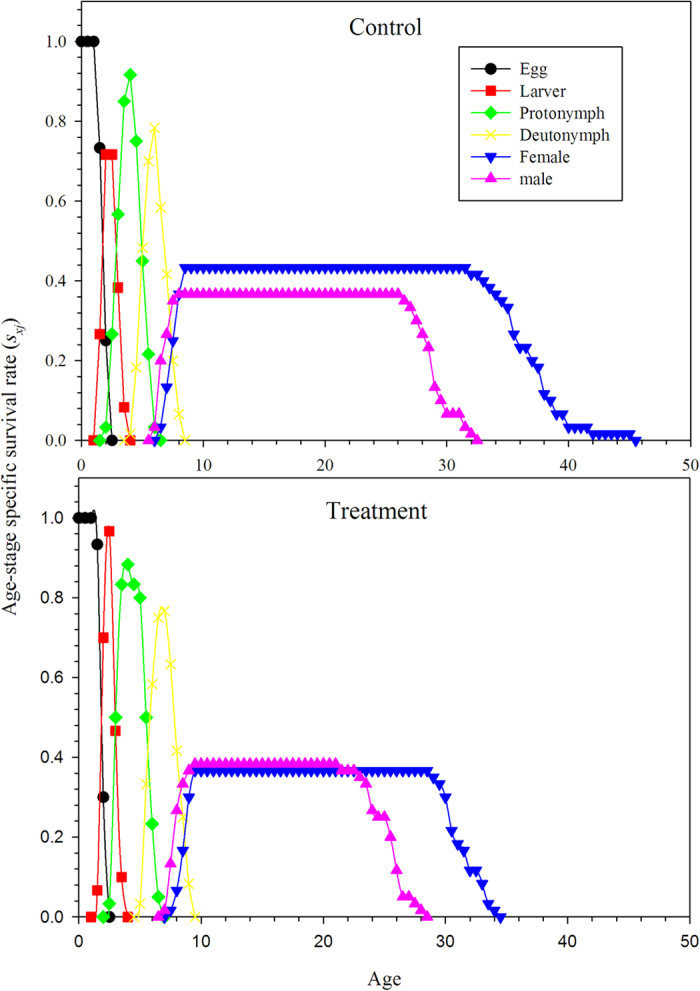
Age-stage survival rate (*s*_*xj*_) of *N. barkeri* in control and treatment. Control means feeding on untreated *F. occidentalis*; Treatment means feeding on *B. bassiana*-treated *F. occidentalis*. Notation is the same as for Figs 2, 3, 4, 5, 6.

**Figure 2:**
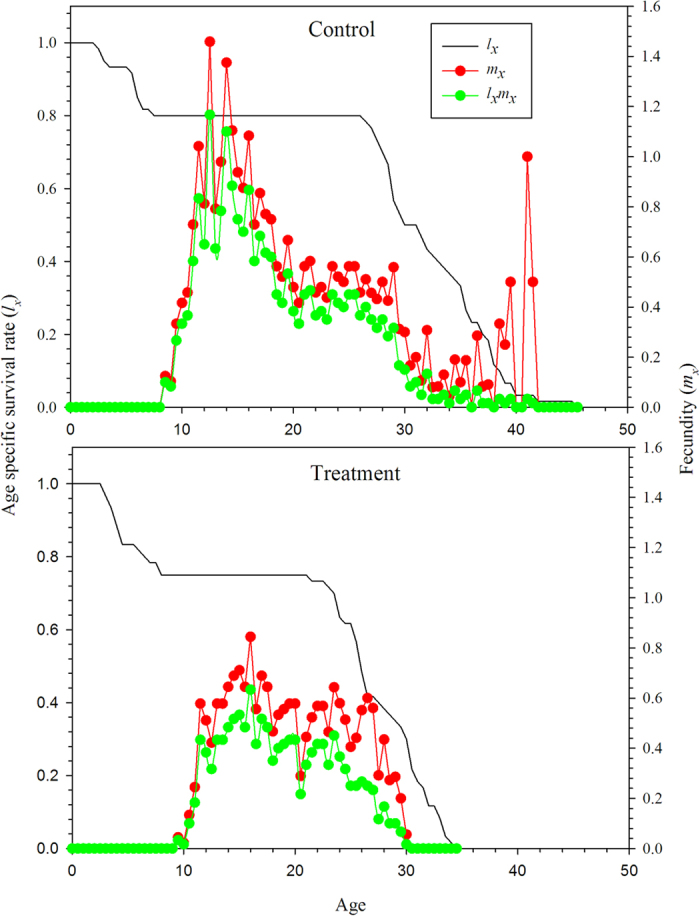
Age-specific survival rate (*l*_*x*_) and fecundities (*m*_*x*_) of *N. barkeri* in control and treatment.

The curves of the age-stage survival rate (*s*_*xj*_) show the probability that an *N. barkeri* egg will survive to age *x* and stage *j* (Fig. 1). The overlap of the stage-specific survivorship curves is the result of the variable developmental rates among individuals^34^. The probability that a newly hatched larva survives to the adult stage was 78.3% in the treatment group, which was lower than the control (83.3%). The curve of the age-stage survival rate (*l*_*x*_) includes all individuals of the cohort and is a simplified version of the *s*_*xj*_ curves (Fig. 2). The age-specific fecundity of the total population (*m*_*x*_) was also plotted in Fig. 2. The curve of *m*_*x*_ shows approximate periodic peaks in reproduction. The highest age-specific reproductive peak occurred at age 12.5 days, with the highest fecundity of 1.5 hatched eggs within 12 h in the control. The reproductive peak occurred at age 16 days, with the highest fecundity of 0.8 hatched eggs within 12 h for the treatment group.

Population parameters were calculated based on data from the entire cohort^35^. The means and standard errors of the intrinsic rate of increase (*r*), finite rate of increase (*λ*), net reproductive rate (*R*^0^) and mean generation time (*T*) are listed in Table 2. Statistical analysis showed that *r*, *λ* and *R*^0^ were lower in the treatment group than the control. Because of the longer developmental time of the pre-adults in the treatment group, *T* was significantly longer than in the control.

**Figure 3:**
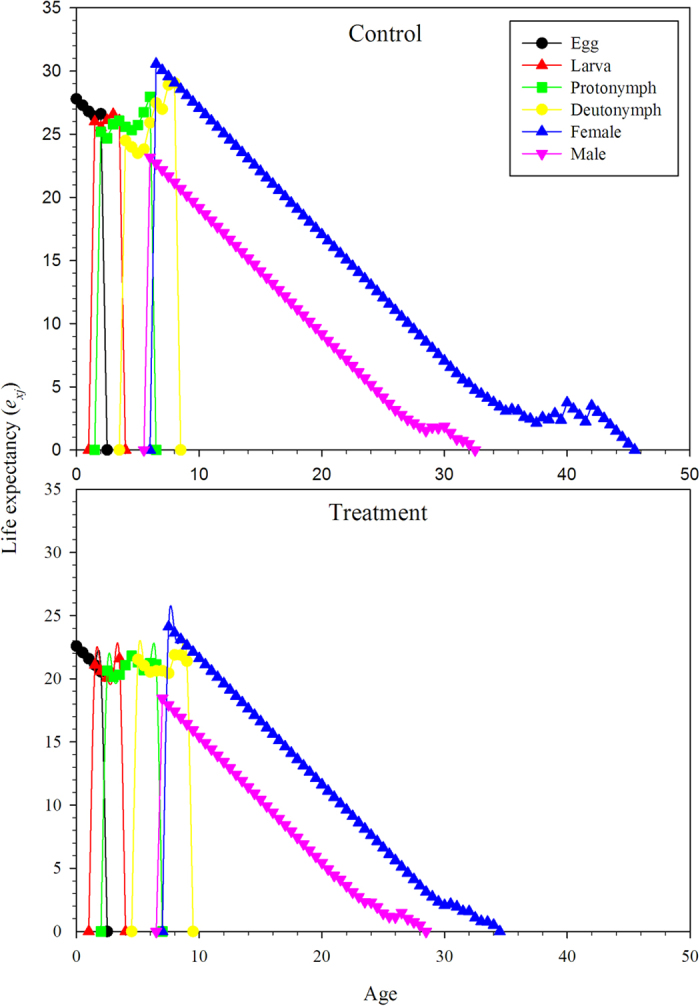
Age-stage-specific life expectancy (*e*_*xy*_) of *N. barkeri* in control and treatment.

**Figure 4:**
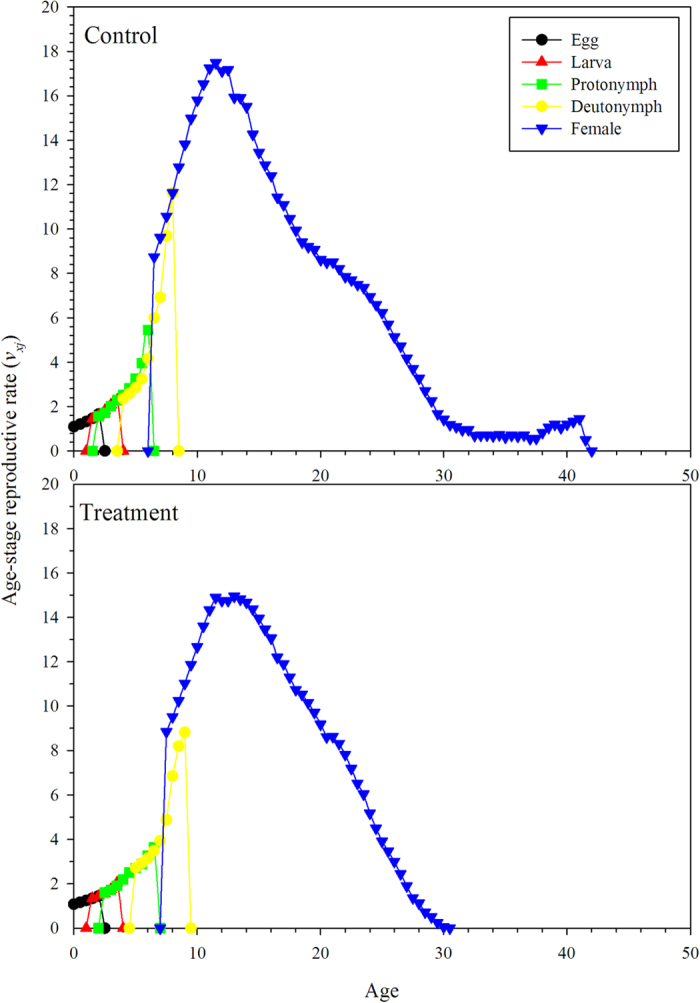
Age-stage-specific reproductive value (*v*_*xy*_) of *N. barkeri* in control and treatment.

The age-stage life expectancy (*e*_*xj*_) is the total time that an individual of age *x* and stage *j* is expected to live after age *x* (Fig. 3). The life expectancy of the *N. barkeri* treatment cohort was shorter than the control. The life expectancy decreased gradually with age. The age-stage-specific reproductive values (*v*_*xj*_) of *N. barkeri* represent the contribution of an individual at age *x* and stage *j* to the future population. The reproductive value increased significantly when *N. barkeri* began to produce hatchable eggs. In the control, the reproductive value increased at age 11.5 days and reached a peak of 17.5. Despite the low fecundity in the treatment group, the peak reproductive value also occurred at age 11.5 days and remained high for 2 days (Fig. 4).

### Predation rate

**Figure 5:**
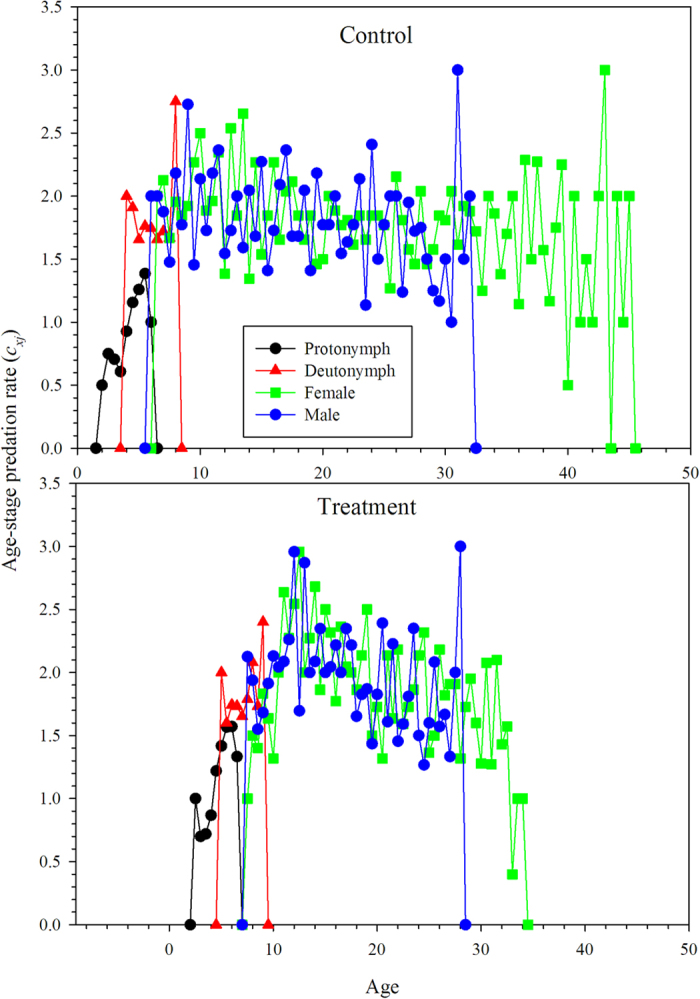
Age-stage, two-sex predation rate (*c*_*xj*_) of *N. barkeri* in control and treatment.

**Figure 6:**
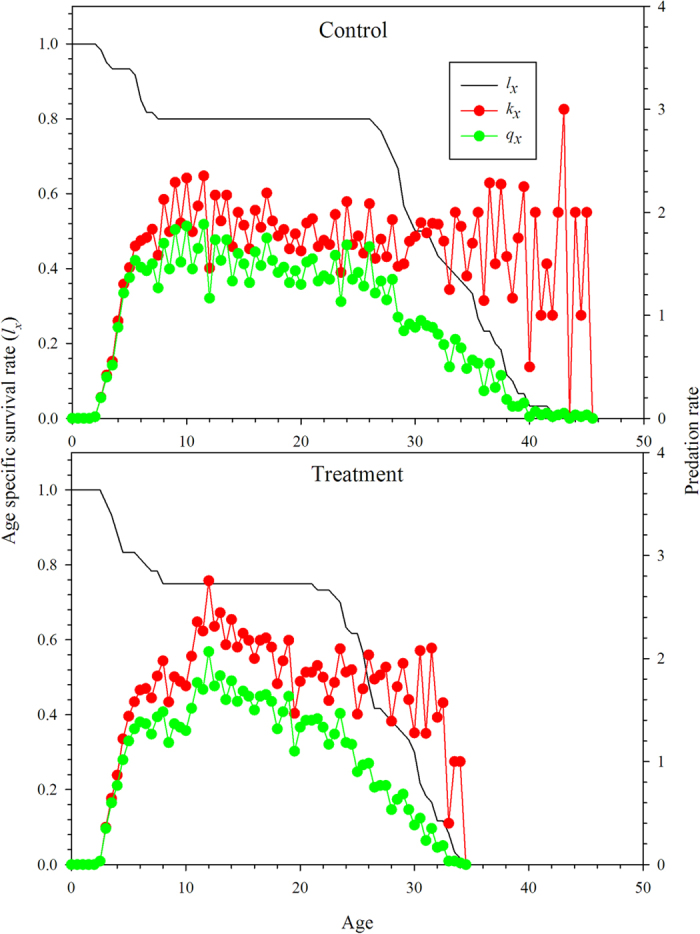
Age-specific survival rate (*l*_*x*_), predation rate (*k*_*x*_), and net predation rate (*q*_*x*_) of *N. barkeri* in control and treatment.

The age-stage predation rate (*c*_*xj*_) of *N. barkeri* fed on *B. bassiana*-treated (treatment) and untreated *F. occidentalis* larvae (control) are shown in Fig. 5. This shows the trend in the age-stage specific mean number of *F. occidentalis* larvae consumed by a predator at age *x* and stage *j*. Using equations (9) and (10), we calculated the age-specific predation rate (*k*_*x*_) and age-specific net predation rate (*q*_*x*_). Both *k*_*x*_ and *q*_*x*_ were calculated by considering the sex and stage differentiation (Fig. 6). When offered *B. bassiana*-treated *F. occidentalis* larvae, the protonymphs and deutonymphs of *N. barkeri* consumed more prey, while both female and male adults lived longer when fed on untreated *F. occidentalis* larvae, and consumed more total prey as adults. By taking survival rates and longevities into account, the net predation rate, C_0_, was 86.0320 when fed on untreated thrips larvae and 70.1384 when fed on *B. bassiana*-treated thrips larvae. However, the finite predation rate of *N. barkeri* was 1.0459 when fed on treated prey, higher than that of *N. barkeri* fed on untreated prey (Table 3).

## Discussion

Fungal biological agents are used to suppress many species of pests; however, they may also negatively affect natural insect enemies through direct infection or indirectly by decreasing the prey population^27^ or affecting the quality of prey^32^. Our previous studies demonstrated that entomopathogenic fungus *B. bassiana* strain SZ-26 showed high toxicity against *F. occidentalis*, but no direct pathogenicity to the predatory mite *N. barkeri*^20^. In the present study, the indirect effects of SZ-26 on *N. barkeri* fed on *B. bassiana*-treated *F. occidentalis* larvae were examined, and the results indicated that biological parameters of the predators were affected. Similar results by Simelane *et al.*^36^, demonstrated that when *Coccinella septempunctata* L. (Coleoptera: Coccinellidae) fed on aphids infected with *Neozygites fresenii* (Nowakowski) (Entomophthorales: Neozygitaceae), the developmental time was significantly longer and the fecundity was lower than *C*. *septempunctata* fed on uninfected aphids. Seiedy *et al.*^32^ also demonstrated that the longevity and fecundity of the predatory mite *Phytoseiulus persimilis* Athias-Henriot (Acari: Phytoseiidae) were adversely affected when fed on *Tetranychus urticae* Koch (Acari: Tetranychidae) treated with *B. bassiana* at 24, 48 and 72 h post-inoculation.

Predatory mites prefer to prey on the first instar of thrips^12^, the first instars develop to second instars within 24–48 h under normal laboratory conditions^37^, so the first instars were supplied to *N. barkeri* as prey every 12 h, and the life table raw data of individual *N. barkeri* were recorded. Using the age-stage, two-sex life table, we accurately and precisely described the survival rate and stage structure, thereby evaluating the sub-lethal effects of *B. bassiana* on *N. barkeri*. From our statistical analysis, the protonymphs and deutonymphs of *N. barkeri* readily fed more on infected *F. occidentalis* larvae than on untreated larvae. Also, the finite predation rate of *N. barkeri* fed on infected prey was higher than that of *N. barkeri* fed on untreated prey. However, the longevity of *N. barkeri* fed on infected prey became shorter, consequently the net predation rate of *N. barkeri* was lower than the *N. barkeri* fed untreated prey.

Our data show that *B. bassiana* has indirect negative effects on *N. barkeri* mediated by their shared host/prey *F. occidentalis*. However, when considering the probable mechanism, it is complicated due to the complex predator-fungus-pest interactions. After having been treated with *B. bassiana* suspension for 12 h, a large number of conidia adhered to the cuticles of the first instars. The majority of conidia germinated and the germ tubes oriented towards the cuticle of the *F. occidentalis* larvae, and some germ tubes penetrated the cuticle of *F. occidentalis* larvae under fluorescence and scanning electron microscopy (SEM) (see Supplementary Fig. S1). Although these infected larvae remained live at this time, their vitality had declined due to fungal penetration and thus they were more vulnerable to predation^38^. Therefore, *N. barkeri* consumed more infected prey than normal prey, perhaps because of the greater catch efficiency of *N. barkeri* on infected *F. occidentalis* larvae, rather than a preference for infected larvae. While they consumed more infected thrips, the two-sex life table parameters of *N. barkeri* were negatively affected. We put forward two possible reasons to account for the results: (1) the poorer nutritional quality of *B. bassiana*-infected thrips could not meet the needs of normal development and reproduction by *N. barkeri*. Huang *et al.*^39^ reported that proteins or saccharides are the essential nutrients for mass production of *N. barkeri*. When *B. bassiana* invades the insect body, it absorbs amino acids from the blood^40^. From our SEM observations, germ tubes of conidia would absorb nutrition from thrips once its cuticle was penetrated. The fungal hyphae would grow further and colonize the host, and the host nutrients were absorbed gradually^41^. Moreover, when offered *B. bassiana*-infected thrips, nearly 50% of the observed dead thrips were partially consumed by *N. barkeri*, further indicating that the quality of the prey was affected by the fungus. When fed on infected thrips, the body size of female *N. barkeri* was smaller than conspecifics fed a diet of untreated thrips. The length and width of female *N. barkeri* adults fed on infected thrips for 15 d were 361.5 and 238.9 μm, which were significantly shorter than those fed on untreated thrips (length: 403.2 μm; width: 296.9 μm) (see Supplementary Table S1); (2) the second possible reason could be because metabolites^42^ or toxins^43^ were produced when the tissues of *F. occidentalis* larvae were invaded by fungal hyphae, which may have harmed the predators. This hypothesis was supported by Agboton *et al.*^25^ and Seiedy *et al.*^32^

Agboton *et al.*^25^ reported that the predatory mite *Typhlodromalus aripo* could consume significant amounts of the entomopathogenic fungus *Neozygites tanajoa*-infected cassava green mite (CGM), *Mononychellus tanajoa,* under laboratory conditions and that the oviposition and survival rate of *T. aripo* were reduced. Onto *et al.*^26^ further showed in screenhouse experiments that the simultaneous presence of *T. aripo* and *N. tanajoa* improved the biological control of the CGM, even though the two agents on the same cassava plants had a negative effect on their respective population growth, such that the *T. aripo* population increased while the *N. tanajoa* population decreased. Our data indicate it is risky for *N. barkeri* when feeding on the *B. bassiana* -infected *F. occidentalis* larvae. This study will help to enhance our understanding about predator-fungus-pest interactions. However, field studies of *B. bassiana* applications in conjunction with *N. barkeri* for biological control of *F. occidentalis* should be conducted, as it remains unknown whether *N. barkeri* behavior, such as search activity, may be reduced under field conditions; our results indicate that *N. barkeri* prefer to prey on relatively less mobile infected thrips. Moreover, if the fungus-infected prey is detrimental to the predator, it might be expected that the latter should have evolved the ability to avoid areas where the fungus is present^30^. Therefore, whether *N. barkeri* will display behavioral avoidance of infected *F. occidentalis* when co-existing with *B. bassiana* and *F. occidentalis* requires further exploration.

## Methods

### Ethics Statement

No specific permissions were required for these locations/activities.

None of the species used in this study are endangered or protected.

### Fungal preparations

The isolate of *B. bassiana sensu lato* SZ-26 was derived from *Ostrinia nubilalis* (Lepidoptera: Pyralidae) collected in Suizhong, Liaoning (2010). Fresh cultures, initiated using conidia inoculum, were maintained, and conidia were produced on Sabouraud dextrose agar (SDA) at 26 ± 1 °C with continuous darkness. The conidial concentrations were determined using a hemocytometer and adjusted with sterile 0.05% Tween-80. Viability of the conidia was confirmed according to the method of Wen *et al.*^44^ and was >90%.

### Predatory mite colony

A colony of *N. barkeri* was obtained from the laboratory of insect natural enemies, Institute of Plant Protection, Chinese Academy of Agricultural Sciences. The stock colony was then cultured on excised kidney bean leaves and supplied *Tyrophagus putrescentiae* (Schrank) (Acari: Acaridae) as prey in plastic boxes (15 cm × 15 cm × 10 cm) with rims. Circular moist sponges (10-cm diameter) were placed at the edges of the boxes to prevent escape. A hole (12-cm diameter) was punched on the lid and covered with a fine mesh to allow for ventilation. The culture boxes were maintained at 25 ± 1 °C, 65 ± 5% RH and L16:D8 h photoperiod in climatic chambers. Cotton silk was placed on the leaf surfaces as an oviposition substrate. The newly laid eggs were collected and transferred into new boxes using a fine brush after 6 hours to allow the emergent larvae to develop in synchrony.

### Western flower thrips colony

A colony of western flower thrips, *F. occidentalis*, was maintained according to the methods described by Liang *et al.*^45^. Briefly, colonies were continuously reared on kidney beans (*Phaseolus vulgaris* L. var. Gonggeizhe) in tube-shaped jars (0.5 L) with snap-on lids. A hole (10-cm diameter) was punched on the lid and covered with a fine mesh to allow for ventilation. The rearing jars were maintained at 26 ± 2 °C, 65 ± 5% RH and L13:D11 h photoperiod in climatic chambers. The thrips at similar development stages were obtained by incubating the adults on fresh plants for oviposition. After 3 days, thrips were removed and we allowed the different stages of *F. occidentalis* to develop in synchrony. The first instar larvae were collected for experimental use.

### Life table study

The experimental units contained two uniform Plexiglas (6 cm × 5 cm × 4 mm) containers. Water-saturated filter paper was placed on the bottom of one piece, with a freshly excised kidney bean leaf added upside down on the filter paper surface. A hole (2.5-cm diameter) was punched on another piece of glass and pressed onto the leaf. A chamber was formed between two pieces of organic-glass as an experimental platform. Sixty eggs of *N. barkeri* were randomly collected from the colony within 6 h. Each egg was transferred carefully into an individual chamber using a fine paintbrush. The experimental units were placed on Petri dishes (20-cm diameter) with moist sponges at the bottom and then placed in a climatic chamber at 25 ± 1 °C, 65 ± 5% RH and L16:D8 h photoperiod. The first instar larvae of *F. occidentalis* were sprayed with a conidia suspension (10^8^ conidia mL^−1^) with a small hand pressure sprayer (2 ml)^38^. After 12 h, the treated thrips were supplied with *N. barkeri* as prey. The units were observed every 12 h until the eggs hatched into larvae. In the preliminary test, each larva developed into a protonymph normally without feeding. Therefore, in our life table study, when the larvae developed into a protonymph, each protonymph was provided 5 *B. bassiana-*treated *F. occidentalis* larvae as prey every 12 h, and each deutonymph was provided 10 *B. bassiana-*treated *F. occidentalis* larvae as prey every 12 h. The survival and development time of each stage were recorded every 12 h. Because the female deutonymphs generally develop females slower than males, the newly emerged males continue to be provided with 10 treated *F. occidentalis* larvae as prey every 12 h. The newly emerged females were paired with young male adults recruited from the colony for mating in individual chambers and provided 20 treated *F. occidentalis* larvae every 12 h. The number of *F. occidentalis* larvae was based on the preliminary feeding test. Survivorship, fecundity, and predation were recorded every 12 h for *N. barkeri*. Because each protonymph, deutonymph and male was housed in an individual chamber, predation was recorded every 12 h for each individual. However, because the adult females and recruited males were housed as pairs in an individual chamber, we ignored the differences between the sexes, and one-half of the daily predation of a pair was assigned to each female as long as both remained alive. If the male died, the total daily predation rate was attributed to the female, and if the female died, the experiment was terminated. The control was the untreated-*F. occidentalis* larvae and was performed as described above.

### Age-stage, two-sex life table data analysis

The life history data of all *N. barkeri* individuals were analyzed based on the age-stage, two-sex life table^46^ with the computer program of TWO-SEX-MSChart^47^. Following the method of Chi and Liu^35^, the survival rate (*s*_*xj*_) (*x* = age, *j* = stage) and fecundity *f*_*xj*_ were calculated. The age-specific survival rate (*l*_*x*_) was then calculated as follows:


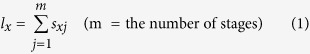


The age-specific fecundity (*m*_*x*_) was calculated as follows:


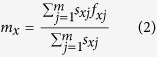


The net reproductive rate (*R*_*0*_) was calculated as follows:


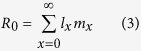


The intrinsic rate of increase (*r*) was estimated according to the Euler–Lotka formula with the age indexed from 0^48^ as follows:


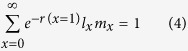


The finite rate (*λ*) was calculated as follows:


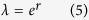


The mean generation time (*T*) was calculated as follows:


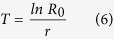


The age-stage-specific life expectancy (*e*_*xy*_) was calculated by using the method of Chi and Su^49^ as follows:


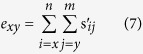


where *s'*_*ij*_ represents the probability that an individual at age x and stage y will survive to age *i* and stage *j*.

The reproductive value (*v*_*xy*_) is defined as the contribution of individuals at age *x* and stage *y* to the future population^50^:


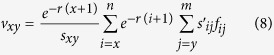


The bootstrap technique was used to estimate the means and standard errors of the population parameters^51^,^52^. Differences between treatment and control were compared using *t*-tests.

### Predation rate analysis

The predation rate data were analyzed based on the computer program of CONSUME-MSChart^53^. The daily predation of all individuals, including males, females, and those dying before adulthood, was used to calculate the age-stage specific consumption rate *c*_*xj*_. This is the mean predation rate of an individual at age *x* and stage *j*. Following the method of Chi and Yang^34^ and Yu *et al.*^54^, the age-specific predation rate (*k*_*x*_) represents the mean predation rate of an individual at age *x* and was calculated as follows:


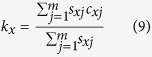


When the age-specific survival rate was taken into consideration, the age-specific net predation rate (*q*_*x*_) was calculated as follows:


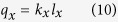


Net predation rate (*C*_*0*_) represents the total number of prey that an average individual predator can kill during its life span and was calculated as follows:


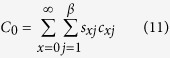


In order to compare the predation potential of different individuals consume the same prey or the same individual consume different preys, Chi *et al.*^55^ suggested using the finite predation rate (*ω*) and was calculated as follows:


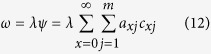


where λ represents the finite rate of an individual with a stable age-stage distribution, *ω* represents the stable predation rate (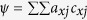), and *a*_*xj*_ represents the proportion that individuals belong to age *x* and stage *j* with a stable age-stage distribution.

The bootstrap technique was used to estimate the means and standard errors of the population parameters^52^. Differences between treatment and control were compared using *t*-tests.

## Additional Information

**How to cite this article**: Wu, S. *et al.* Feeding on *Beauveria bassiana*-treated *Frankliniella occidentalis* causes negative effects on the predatory mite *Neoseiulus barkeri*. *Sci. Rep.* **5**, 12033; doi: 10.1038/srep12033 (2015).

## Supplementary Material

Supplementary Information

## Figures and Tables

**Table 1:** Developmental time, longevity and mean fecundity of *N. barkeri* in control and treatment.

Stage	n	Control Developmental time (d) (mean ± SE)	n	TreatmentDevelopmental time (d) (mean ± SE)
Egg	60	1.99 ± 0.06a	60	2.12 ± 0.05a
Larva	57	1.08 ± 0.05a	57	1.15 ± 0.04a
Protonymph	56	2.11 ± 0.05b	49	2.46 ± 0.08a
Deutonymph	48	1.84 ± 0.06b	45	2.34 ± 0.06a
Preadult	48	7.25 ± 0.10b	45	8.39 ± 0.10a
Adult longevity				
Female	27	29.46 ± 0.55a	23	22.86 ± 0.38b
Male	23	22.34 ± 0.32a	24	17.39 ± 0.30b
Apop	27	2.54 ± 0.12a	23	2.50 ± 0.10a
Tpop	27	10.13 ± 0.19b	23	11.25 ± 0.13a
Mean fecundity				
Female	27	52 ± 2a	23	38 ± 1b

Values followed by the different lowercase letters within a row are significantly different using *t*-tests (*P* < 0.05). APOP represents adult pre-oviposition period; TPOP represents total pre-oviposition period.

**Table 2:** Population parameters of *N. barkeri* in control and treatment.

Parameter	Control (mean ± SE)	Treatment (mean ± SE)
Intrinsic rate of increase, *r* (d^−1^)	0.1896 ± 0.0108a	0.1461 ± 0.0109b
Finite rate of increase, *λ* (d^−1^)	1.2088 ± 0.0130a	1.1572 ± 0.0170b
Net reproductive rate, *R*^0^ (offspring)	22.4833 ± 3.3773a	13.4634 ± 2.3000b
Mean generation time, *T* (d)	16.3684 ± 0.2580b	17.7014 ± 0.2660a

Values followed by the different lowercase letters within a row are significantly different using *t*-tests (*P* < 0.05).

**Table 3:** Predation rates of *N. barkeri* in control and treatment.

Stage	Predation rate (preys/predator)	
	Control(mean ± SE)	Treatment (mean ± SE)
Larva	0	0
Protonymph	3.93 ± 0.17b	5.88 ± 0.22a
Deutonymph	6.71 ± 0.26b	8.49 ± 0.26a
Preadult	10.71 ± 0.35b	14.31 ± 0.4a
Adult	96.02 ± 2.74a	78.36 ± 1.94b
Net predation rate, *C*_*0*_	86.0320 ± 5.8587a	70.1384 ± 5.1868b
Stable predation rate, *ψ*	0.7862 ± 0.0250b	0.9038 ± 0.0288a
Finite predation rate, *ω*	0.9503 ± 0.0328b	1.0459 ± 0.0381a

Values followed by the different lowercase letters within a row are significantly different using *t*-tests (*P* < 0.05).
