# Supplementary material for: Feeding on Beauveria bassiana-treated Frankliniella occidentalis causes negative effects on the predatory mite Neoseiulus barkeri
Source: Sci Rep. 2015 Jul 8;5:12033. doi: 10.1038/srep12033 (PMC4495565; doi:10.1038/srep12033)
Supplement: Supplementary Information [file srep12033-s1.pdf]

Supplementary Information

Feeding on *Beauveria bassiana*-treated *Frankliniella occidentalis* causes negative effects on the predatory mite *Neoseiulus barkeri*

Shengyong Wu[1], Yulin Gao[1], Xuenong Xu[1], Dengjie Wang[1], Juan Li[1], Haihong Wang[1], Endong Wang[1] and Zhongren Lei[1*]

[1]State Key Laboratory for Biology of Plant Diseases and Insect Pests, Institute of Plant Protection, Chinese Academy of Agricultural Sciences, Beijing 100193, P.R. China

*Corresponding author Email: zrlei@ippcaas.cn (ZL)

Supplementary Table S1 The body length and width of female *N. barkeri* feeding on *B. bassiana* -infected *F. occidentalis* larvae (treatment) and untreated larvae (control)

	Length (μm) (mean±SE)	Width (μm) (mean±SE)
Treatment	361.5±4.06b	238.9±5.26b
Control	403.2±2.08a	296.9±2.37a
$F_{1, 38}$	3.63	3.15
t	9.13	10.06
P	<0.001	<0.001

Values followed by the different lowercase letters within a column are significantly different using *t*-tests (P <0.05).

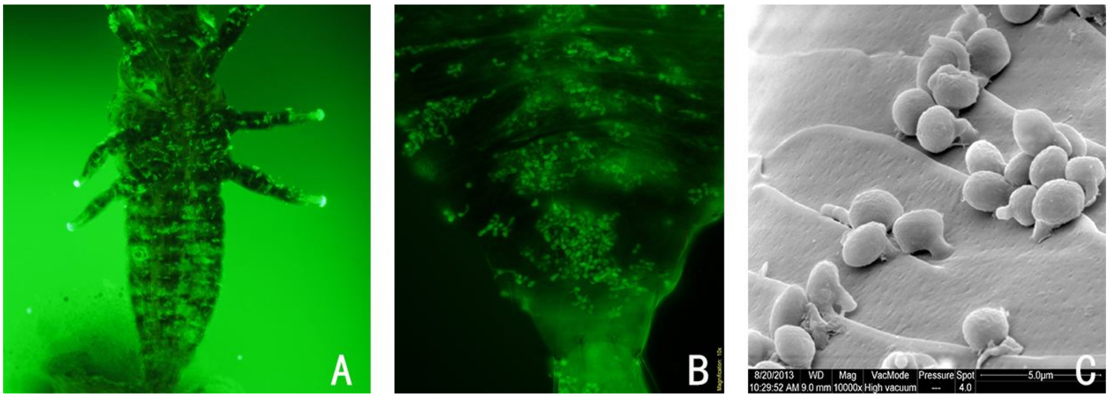

Supplementary Fig. S1 Germination and infection of *B. bassiana* conidia on the cuticle of first instar larvae of *F. occidentalis* 12 h after treatment with *B. bassiana* using fluorescence and scanning electron microscopy. (A) conidia fluoresced green on the cuticle of *F. occidentalis* at 40× magnification; (B) germinated conidia on the cuticle of *F. occidentalis* at 100× magnification; (C) germ tube penetrating the cuticle
